# Assessment of epicardial adipose tissue volume and carotid intima-media thickness in children with primary arterial hypertension by magnetic resonance imaging

**DOI:** 10.2478/raon-2025-0030

**Published:** 2025-05-14

**Authors:** Nina Schweighofer, Natasa Marcun Varda, Primoz Caf, Mitja Rupreht, Vojko Kanic, Petra Povalej Brzan

**Affiliations:** Department of Radiology, University Medical Centre Maribor, Maribor, Slovenia; Department of Pediatrics, University Medical Centre Maribor, Maribor, Slovenia; Department of Cardiology, University Medical Centre Maribor, Maribor, Slovenia; Medical Faculty, University of Maribor, Maribor, Slovenia; Faculty of Electrical Engineering and Computer Science, Maribor, Slovenia

**Keywords:** epicardial adipose tissue, arterial hypertension, magnetic resonance imaging, children, atherosclerosis, intima-media thickness

## Abstract

**Background:**

Epicardial adipose tissue (EAT) is a biologically active visceral brown adipose tissue, which is irregularly distributed across myocardium. It has emerged as a potential modifiable cardiometabolic biomarker in adults, demonstrating pro-inflammatory properties with involvement in subclinical atherosclerosis. The increased thickness of the inner two layers of the carotid artery wall (intima and media) in childhood can pose as a risk of the development of atherosclerotic disease and its complications in adult life, representing additional potential biomarker. The purpose of our study was to evaluate a relation between EAT volume (EATV) and carotid intima-media thickness (cIMT) in children and adolescents who have been diagnosed with primary arterial hypertension (AH), utilizing magnetic resonance imaging (MRI).

**Patients and methods:**

The study included 72 children and adolescents, half of them had an established diagnosis of primary AH and the other half were matched healthy controls. The EATV and cIMT measurements were compared between the two groups and correlated with clinical, anthropometric and functional parameters.

**Results:**

Children diagnosed with AH exhibited a significantly higher EATV (16.5 ± 1.9 cm^3^ *vs*. 10.9 ± 1.5 cm^3^; t = –13.815, p < 0.001) and higher cIMT (0.7 [0.2] mm *vs*. (0.4 [0.1) mm]; U = 54, p < 0.001) compared with their healthy counterparts. EATV demonstrated a significant correlation with cIMT.

**Conclusions:**

Increased EATV and cIMT were found with MRI in hypertensive children compared to their healthy counterparts. EATV demonstrated a stronger association with hypertension than cIMT. EATV emerged as an independent predictor of cIMT.

## Introduction

Primary arterial hypertension (AH) is characterized by elevated blood pressure (BP) without a specific identifiable cause. In children and adolescents, the diagnosis of AH is based on BP values relative to the percentile distribution in healthy children. Blood pressure levels at or above the 95th percentile must be measured on at least three separate visits.^1^ In the pediatric population, AH is rapidly increasing, with a global prevalence between 13% and 18%.^1,2^ Hypertensive youth are more likely to develop hypertension in adulthood and have a higher probability of experiencing target organ damage, including hypertensive heart disease, atherosclerosis, and stroke.^3,4^ Atherosclerosis is a systemic disease, which impairs normal function of mainly large and medium-sized arteries (coronary, carotid, cerebral, and renal).^5^

Early vascular dysfunction can be identified in childhood, and its advancement is influenced by exposure to various risk factors, such as obesity along with metabolic disorders, hypertension, and chronic inflammatory conditions.^6^ The pathophysiologic process includes both the media and intima layers of vascular wall, resulting in progressive stiffening of arterial trunks, progressive narrowing at common sites, and risk of thromboembolic complications.^7^ Early atherosclerotic lesions may already be present at a young age with subsequent evolvement into clinically evident cardiovascular disease (CVD).^8,9^ This progression continues throughout adult life, where fatty and fibrous tissue cells can present a significant portion, up to 30%, of the aortic intima, even in individuals who were otherwise considered healthy.^10^

The increased thickness of the inner two layers of the carotid artery wall (intima and media, cIMT), serves as a one of the indicators of early atherosclerosis and can be utilized to assess cardiovascular (CV) risk.^11,12^ In pediatric population, elevated intima-media thickness is proposed to be an adaptation of the vascular wall to increased pressure of blood in the absence of atherosclerotic lesions.^13,14^ Consequently, cIMT values increase relating to physiological processes along with age, male sex, sexual maturation, and certain ethnic groups.^10,15,16^ However, an elevation beyond an age specific cut-off value could signify vascular remodeling in response to atherosclerotic risk factors, contributing to an increased incidence of cardiovascular events in adulthood.^17,18^ A recent study concluded that increased cIMT in childhood can pose as a risk of the development of atherosclerotic disease and its complications in adult life.^19^

The measurement of cIMT can be conducted through various methods. The most common and clinically employed approach is the ultrasound (US) examination of carotid arteries with advantages such as time efficiency and cost-effectiveness with an important limitation of interreader variability. Magnetic resonance imaging (MRI) is increasingly utilized for in-depth evaluation of carotid arteries due to its inherent high soft tissue resolution.^20-22^ The assessment of cIMT using high-resolution MRI has already been suggested as an alternative non-invasive surrogate marker of cardiovascular disease. Potential advantages of MRI over US are the ability to evaluate concentric heterogeneity and potentially including the arterial adventitia in the measurement of wall thickness.^23^

Several studies proposed a pathophysiologic relation between cIMT and epicardial adipose tissue (EAT). EAT is biologically active visceral brown adipose tissue, which is irregularly distributed across almost three-quarters of the myocardium, mainly on the right ventricle and the lateral aspect of the right atrium. EAT also surrounds coronary arteries. There is no fascia or connective tissue separating EAT and cardiomyocytes, which implies a close interaction between these two structures.^24,25^ EAT consists of adipocytes, stromal cells, and inflammatory cells, which secrete bioactive molecules with a potential for modulating cardiac and vascular function and morphology. Increasing evidence supports the conclusion that EAT could act as a promoter of early atherosclerotic disease.^26,27^ Furthermore EAT is closely related to cIMT and early cardiac dysfunction in obese adolescents.^28^

In our previous work, we demonstrated that children and teenagers with primary AH exhibited greater amount of EAT compared to healthy controls.^29^ To the best of our knowledge, no studies have investigated the relation between the volume of EAT (EATV) and cIMT in hypertensive pediatric patients utilizing MRI. Therefore, the purpose of this study was to determine a possible link between EATV and cIMT in hypertensive children, assessed with MRI, compared to their healthy counter parts.

## Patients and methods

### Study population

The study included 72 Caucasian children and adolescents of healthy weight range. All of the included children were from Slovenia and were elementary and high school students. The same cohort of patients has already been studied to assess MRI for characterizing EAT and its correlation to hypertension.^29^ The initial group comprised 36 individuals diagnosed with primary AH.^30^ The children were diagnosed and treated according to the European Society of Hypertension (ESH) guidelines for the management of high blood pressure in children and adolescents (2016).^30^ The other group served as a control, consisting of healthy participants who matched the study group in terms of age, sex, weight, height, and body surface area (BSA). The children in the control group volunteered to take part in our study. They were introduced by their primary health care practitioners. The children had no known medical history of an elevated blood pressure. All of the included participants were regularly physically active (at least 3 times a week for at least 2 hours). Subsequently, a comparison was made between the two groups.

### Ethics, consent and permissions

The study received approval from the National Medical Ethics Committee responsible for assessing the ethical integrity of medical practices and medical research in Slovenia (0120-145/2020/7). All participants gave their informed assent for inclusion in the study. Furthermore, informed consent was also obtained from their parents.

### Clinical assessment

Measurements of height, weight, hip, and waist circumference were conducted. The BMI was calculated as weight divided by squared height. BSA was determined according to a variation of the DuBois formula. Blood pressure values were determined with the use of an identical automatic blood pressure measurement device (Omron M2, Omron Healthcare, Kyoto, Japan) in all of the participants, with three readings taken 5 minutes apart after a minimum of 10 minutes of rest. The readings were taken in the morning with an adequately sized cuff. The average value of all readings was recorded.

Diagnosis of primary AH was made according to average systolic or diastolic blood pressure values exceeding the 95th percentile values based on age, sex, and height-specific percentile tables. For adolescents aged 16 years or older, the recorded values of blood pressure had to be higher than 140 mmHg for systolic and 90 mmHg for diastolic threshold on 3 separate visits. Patients were diagnosed based on the ESH guidelines for the management of high blood pressure in children and adolescents from the year 2016.^30^ Since the diagnostic criteria for hypertension in children and adolescents have remained unchanged, the inclusion criteria in our study are also in alignment with the new 2023 ESH guidelines.^31^

To validate the diagnosis of AH, all of the 36 hypertensive patients underwent continuous monitoring of arterial blood pressure for one day period (charts from the article of Soergel *et al*. served as the reference range).^32^ Exclusion criteria included confirmed secondary causes for arterial hypertension (AH), according to the ESH guidelines for the management of high blood pressure in children and adolescents.^30^ All children and teenagers with contraindications for MR imaging (claustrophobia, individuals with certain types of implants etc.) were excluded.

### Imaging acquisition

The MRI acquisition and analyses of the EAT were already presented in our previous work.^29^ In brief, all of the included children underwent MR imaging of the heart and carotid arteries on a 1.5 Tesla MR system Siemens Magnetom Sola imaging device (Siemens AG, Erlangen, Germany). After obtaining the scout images, long-axis and short-axis views were finalized. Eight to twelve short-axis views covering an entire left and right ventricle including every visual part of EAT were acquired with the purpose of anatomical evaluation of the heart. Images were electro-cardiogram gated and taken in a retrospective fashion during an end-expiratory breath-hold. To assess functional parameters, electro-cardiogram gated 2-chamber and 4-chamber cine images were obtained utilizing a segmented steady-state free-precession (time to echo / time of repetition 1.12/6.7 ms, temporal resolution of 35 ms, flip angle of 54°, slice thickness of 8 mm, interslice gap of 2 mm).

**FIGURE 1.**
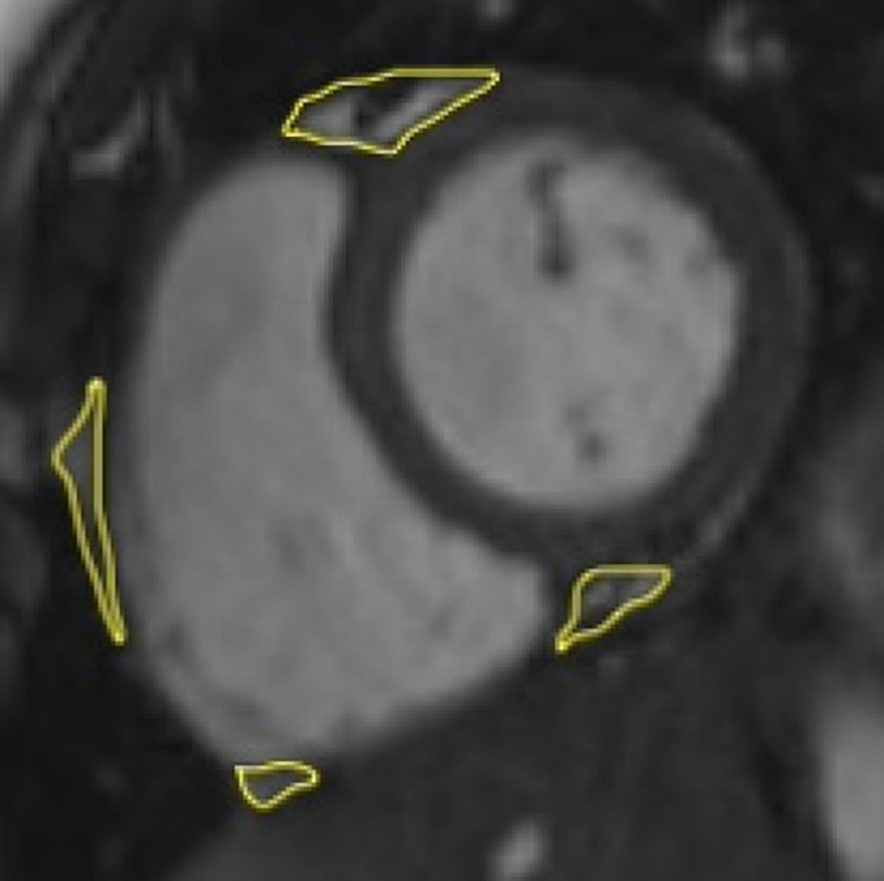
Cine segmented steady-state free-precession MRI of the heart in 14-years-old boy diagnosed with arterial hypertension. Contours of epicardial adipose tissue (EAT) are outlined by hand on end diastolic short-axis image. The EAT volume (EATV) is 14.89 cm^3^.

Imaging analysis was carried out by the aid of software programe Syngo.via (Siemens AG, Erlangen, Germany). EAT contours were outlined by hand on a stack of end diastolic short-axis images. The contoured area on each one of short axis images was later automatically multiplied by 8 mm (the thickness of the image slice) to determine the absolute EATV in the unit of cubic centimeter (Figure 1).

**FIGURE 2.**
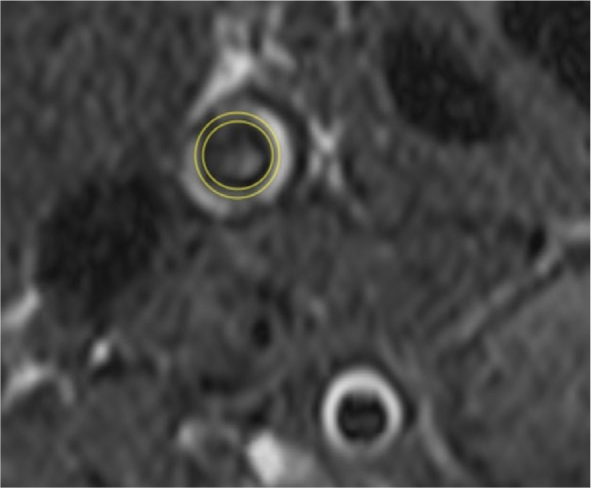
The MR imaging of the common right carotid artery. T1-weighted dark blood fast-spin echo pulse sequence demonstrated adequate soft tissue contrast. The intimamedia thickness of the vessel wall is outlined.

**FIGURE 3.**
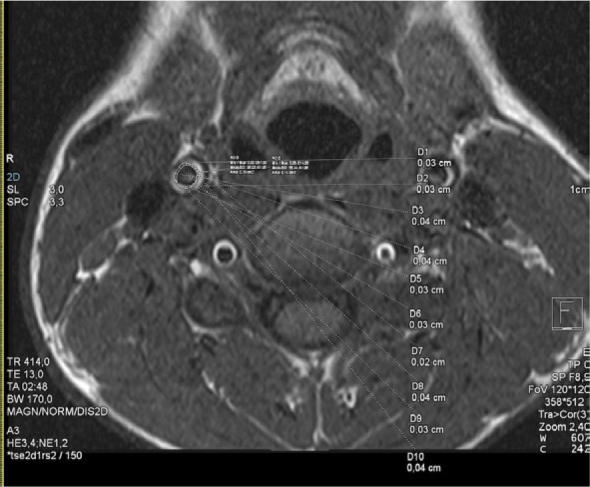
The MR imaging of the right common carotid artery. The measurements of carotid intima-media thickness (cIMT) in a healthy 14-year-old boy. The calculated average cIMT was 0.3 mm.

MR imaging of the carotid arteries was performed on the same 1.5 Tesla MR system. First, time-of-flight (TOF) angiography encompassing both the left and right carotid arteries was obtained to precisely locate the left and right carotid bifurcation. The TOF data were later utilized to position coronal and axial planes in T1-weighted dark blood fast-spin echo pulse sequence, covering the 20 mm of the common carotid artery beneath the bifurcation and the carotid bifurcation on both sides. The imaging parameters were: time to echo 13 ms, time of repetition 414 ms, slice thickness 3 mm). cIMT was measured on both sides, 10 mm below the bifurcation on 10 different equally positioned measurement spots in the carotid wall, and the average value of all readings was recorded (Figures 2, 3). MR derived assessment of EAT and IMT were analyzed by two radiologists (NS (resident) and PC (consultant)), blinded.

### Statistical analysis

The data were expressed either as mean value (MV) ± standard deviation (SD) or as median (M) with interquartile range (IQR) in parameters with non-normal distribution. To assess data distribution, the Shapiro–Wilk normality test was used.

Body mass index (BMI) and Body surface area (BSA) were used to assess body composition. BSA was assessed using a modified version of the DuBois and DuBois formula BSA (m^2^) = 0.007184 × [weight (kg)^0.425^ × height (cm)^0.725^].^33^

Intra-observer reproducibility for EATV and cIMT were evaluated using the intraclass correlation coefficient (ICC) via a two-way mixed effects model. Group comparisons between normotensive and hypertensive groups, based on data distribution, utilized unpaired, two-tailed Student’s t-test and nonparametric Mann–Whitney U test. Fisher’s exact test determined sex differences. Spearman’s Rho test and linear regression analysis were utilized to recognize correlates of EAT volume, and cIMT between groups.

Correlations between anthropometric and morphological parameters with EAT thickness, and cIMT were explored using nonparametric Spearman’s Rho test and linear regression analysis. Receiver operating characteristic curve (ROC) analysis, with area under the curve (AUC) calculation, identified the best parameter for predicting hypertension. The Youden index determined the optimal threshold, maximizing sensitivity and specificity. Positive predictive value (PPV) and negative predictive value (NPV) were also computed.^34^ Linear regression aimed to establish a correlation between EATV and cIMT adjusted for age, sex, BSA and hypertension. The Akaike Information Criterion (AIC) estimated prediction error and model quality. A significance level of p < 0.05 was considered statistically significant. The R programming language (2022, Vienna) was employed for statistical analysis.^35^

## Results

### Study population, clinical assessment and body composition

From 72 participants, comprising 52 were boys and 20 were girls aged between 12 and 19, all within a healthy weight. The first group comprised 36 individuals diagnosed with hypertension, with 27 (75.0%) boys and 9 (25.0%) girls, with an average age of 15.2 ± 1.72 years of age. The second group included 36 healthy children, including 25 (69.4%) boys and 11 (30.6%) girls, with an average age of 15.3 ± 2.2 years of age. The participants in both groups were matched in average age, BMI and BSA. These data mirror our study of the same participant group as referenced in.^29^ Detailed information about anthropometric and body composition parameters are presented in the Table 1 and cardiac morphology and function parameters are presented in the Table 2 of the referenced article.

**TABLE 1.** Comparing epicardial adipose tissue volume and intima-media thickness between groups

	All (N = 72)	Children with AH (N = 36)	Healthy children (N = 36)	Test value (p-value)
**Variable**	**MV ± SD / M (IQR)**	**MV ± SD / M (IQR)**	**MV ± SD / M (IQR)**	
EATV (cm^3^)	13.7 ± 3.3	16.5 ± 1.9	10.9 ± 1.5	*t* = -13.815 (< 0.001)*
cIMT (mm)	0.5 (0.3)	0.7 (0.2)	0.4 (0.2)	U = 54 (< 0.001)*

1 AH = arterial hypertension; cIMT = carotid intima-media thickness; EATV = epicardial adipose tissue volume; IQR = interquartile range; M = median; MV = mean value; SD = standard deviation; *t =* Student’s *t*-test; U = Mann–Whitney U test;

* denotes significance at 99% level, respectively

**TABLE 2.** Regression models for predicting carotid intima-media thickness (cIMT) based on age, sex, body surface area (BSA) and the presence of hypertension

	Model 1 β (SE)	Model 2 β (SE)	Model 3 β (SE)
**Constant**	**-0.221 (0.141)**	**-0.218 (0.124)**	**-0.144 (0.114)**
Age	0.011 (0.008)	0.011 (0.008)	
Gender (female)	0.001 (0.033)		
BSA	0.260 (0.084)*	0.258 (0.074)*	0.313 (0.064)*
Hypertension (yes)	0.273 (0.026)*	0.273 (0.026)*	0.267 (0.026)*
R-squared	0.702	0.702	0.698
Adjusted R-squared	0.684	0.698	0.684
Number of observations	72	72	72

1 β (SE) = regression coefficient (standard Error); R-squared = proportion of variance in the dependent variable explained by the model; adjusted R-squared = Proportion of variance explained by the model, adjusted for the number of predictors;

* p-value < 0.01

### Parameters of cardiac morphology and carotid intima-media thickness (cIMT)

Comparison of anatomical and function parameters between children and teenagers in the hypertensive and healthy control group revealed significant differences. The EATV was notably larger in group with arterial hypertension (16.5 ± 1.9 cm^3^) in comparison with the healthy control group (10.9 ± 1.5 cm^3^) (p < 0,001). Additionally, a significant difference was noted in cIMT between children with arterial hypertension (0.7 (0.2) mm) and their healthy counterparts (0.4 (0.1) mm) (p<0,001) (Table 1).

### Intra-observer reproducibility

The intra-observer reproducibility of measurements for EATV and cIMT was evaluated using the intraclass correlation coefficient. The ICC for the volume of EAT was 0.91 (95% CI, 0.86–0.94), indicating excellent reproducibility. The ICC for cIMT was 0.86 (95% CI, 0.74–0.92), pointing towards good reproducibility. Overall, these results suggest good to excellent reproducibility for EATV and cIMT measurements.

### Associations of cIMT with anthropometric, morphologic and functional parameters

cIMT demonstrates a good reciprocity with BSA (rho = 0.468, p < 0.001) and age (rho = 0.260, p =0.027) of the study group. No significant difference in cIMT was observed between boys and girls (U = 414.500, p = 0.179). The group of children diagnosed with AH exhibited notably higher cIMT than children in normotensive group.

Table 2 presents the multivariate regression models (the regression coefficients (β) and their standard errors (SE)) predicting carotid intimamedia thickness based on BSA and hypertension corrected for gender and age. Each coefficient represents the expected change in cIMT associated with a one-unit change in the corresponding predictor variable, holding all other variables constant. Model 1 is a full model that includes all predictors. Model 2 excludes gender as it was not significanty important in Model 1. The R-squared value remains 0.702, indicating that excluding gender did not reduce the model’s explanatory power. Model 3 additionally excludes age. A one-unit increase in body surface area (BSA) is associated with a 0.313 unit increase in cIMT, and the presence of hypertension is associated with a 0.267 unit increase in cIMT. The R-squared value of 0.698 indicates that approximately 70% of the variance in cIMT is explained by this final model, while the adjusted R-squared value of 0.684 accounts for the number of predictors in the model. The R-squared value shows a minimal decrease in explanatory power compared to Models 1 and 2.

### Can hypertension be predicted based on the epicardial adipose tissue volume (EATV) or carotid intima-media thickness (cIMT)?

**FIGURE 4.**
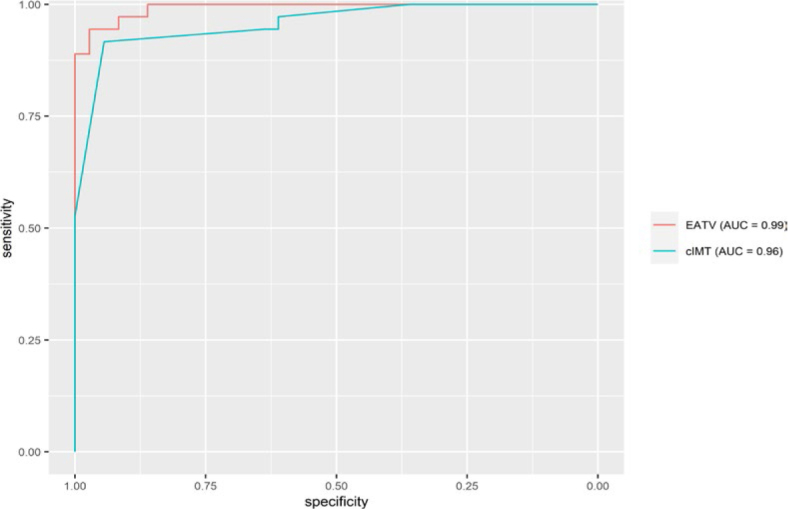
Receiver operating characteristic curve (ROC) and area under the curve (AUC) values were used to assess the performance of potential predictors for hypertension. cIMT = carotid intima-media thickness; EATV = epicardial adipose tissue volume

The optimal prediction variable was assessed using ROC curves and AUC. Both volume of EAT and cIMT demonstrated significant discriminatory capacity between the group of children with AH and healthy control group, EATV emerged as more effective predictor of hypertension in our study group (AUC = 0.99, 95% CI 0.98–0.99), exhibiting a sensitivity of 94.4% and specificity of 97.2%. The positive predictive value of the volume of EAT was 97.1% and negative predictive value was calculated at 94.6%. Further details are presented in Figure 4 and Table 3.

**TABLE 3.** Predictors of hypertension

Predictor	AUC (95% CI)	Threshold	Sen	Spec	PPV	NPV
EATV	0.992 (0.981, 0.992)	13.545	0.944	0.972	0.971	0.946
cIMT	0.958 (0.917, 0.958)	0.550	0.917	0.944	0.943	0.919

1 AUC = area under the curve; CI = confidence interval; cIMT = cardiac intima-media thickness; EATV = epicardial adipose tissue volume; NPV = negative predictive value; PPV = positive predictive value; Sen = sensitivity; Spec = specificity

#### Can cIMT be predicted based on the EATV?

We conducted linear regression analysis to explore the relationship between EATV and cIMT. In the initial model (Model 1), we predicted cIMT using EATV while adjusting for age, gender, body surface area (BSA), and hypertension. Gradually, we eliminated non-significant factors, resulting in the final model (Model 2), which includes only EAT volume as an independent variable. Remarkably, this simplified model explains 82% of the variability in cIMT (R-squared = 0.82, AIC = –150.7). Refer to Table 4 for detailed coefficients and statistical information.

**TABLE 4.** Regression models for predicting cardiac intima-media thickness (cIMT) based on age, gender, body surface area (BSA), epicardial adipose tissue volume (EATV) and the presence of hypertension

	Model 1 β (SE)	Model 2 β (SE)
Constant	-0.285 (0.110)	-0.175 (0.042)
EATV	0.050 (0.007)*	0.053 (0.003)*
Age	-0.001 (0.006)	
Gender (female)	0.014 (0.025)	
BSA	0.087 (0.070)	
Hypertension (yes)	0.008 (0.045)	
R-squared	0.822	0.818
Adjusted R-squared	0.809	0.815
Number of observations	72	72

1 β (SE) = regression coefficient (atandard error); R-squared = Proportion of variance in the dependent variable explained by the model; Adjusted R-squared = Proportion of variance explained by the model, adjusted for the number of predictors;

* p-value < 0.01

## Discussion

EAT and cIMT are emerging as biomarkers, related to the AH and atherosclerosis in both adults and children.^36–39^ However, its prognostic and clinical relevance in the pediatric population remains unclear. cIMT represents another potential biomarker for the risk of atherosclerotic disease. Several studies have examined the relationship among blood pressure values, EAT, cIMT and cardiac remodeling in obese children and came to a conclusion that obese children have higher thickness of EAT and cIMT and higher probability for developing AH in comparison with healthy controls.^38,39^ Nevertheless, data on normal-weight children is scarce, and previous studies have often used ultrasound to measure EAT thickness rather than quantifying EATV with MR imaging. Therefore, the aim of our study was to investigate whether there is a correlation between EATV and cIMT, utilizing the MRI, in hypertensive children with the healthy weight.

Key findings include:

(1) Increased EATV and cIMT in hypertensive children compared to their healthy counterparts. Notably, EATV demonstrated a stronger association with hypertension than cIMT.
(2) Elevated EATV showed a good correlation with increased cIMT in hypertensive children.
(3) EAT volume emerged as an independent predictor of cIMT in our study population.

EAT is a biologically active fat depot that secretes various adipokines, each known for its influence on the pathophysiological processes of cardiovascular disease and early atherosclerosis. The mentioned biologically active molecules include pro-inflammatory molecules (tumor necrosis factor alpha, adiponectin, interleukin, leptin and plasminogen activator inhibitor 1). These adipokines have the potential to negatively impact cardiovascular health and contribute to early atherosclerosis through initiation of subclinical inflammation process in the arterial wall.^40,41^

A possible hypothesis is that the increased EAT volume, influenced by both genetic and environmental factors, leads to heightened production of vasoactive peptides and pro-inflammatory mediators.^42^ This process could activate the renin-angiotensin system and launch an inflammatory environment, pathophysiological mechanisms could lead to an elevation of arterial blood pressure, and potentially contribute to early onset atherosclerosis.

EAT might cause an activation of the autonomic nervous system in the heart by secreting free fatty acids, resulting in raised systemic blood pressure and leading to myocardial hypertrophy and dysfunction.^40^ Moreover, pro-inflammatory and lipotoxic cytokines have the potential to diffuse directly into the cardiomyocytes, leading to hypertrophy and dysfunction of the myocardium. Additionally, plasma proatherogenic cytokines such as interleukin-6, monocyte chemoattractant protein 1, cluster of differentiation-36, and fatty acid transporter 4 could inflict damage on the vascular layers, particularly the intima and media, thereby promoting coronary and potentially peripheral atherosclerosis, including the atherosclerosis of carotid arteries, which can be estimated trough measurement of the cIMT.^43,44^

EAT could have an important role in the development of atherosclerosis and cardiovascular events, therefore positioning itself as a potential therapeutic target.^45,46^ EAT is currently being investigated as a target for gene therapy. The fibroblast growth factor 21 has been proposed as a possible therapeutic agent for several metabolic diseases (fatty liver disease and diabetes (type 2)).^47^

All of the mentioned pathophysiological processes appear to be interconnected, but the exact mechanisms causing primary AH and atherosclerosis in childhood are not yet fully understood. Increased EAT volume and elevated cIMT in children with AH are probably a result of complex interaction between environmental, genetic, and epigenetic factors. It has to be taken into account that higher cIMT values in hypertensive children are to a certain extent consequence of an adaptation of the vascular wall to hemodynamic effects caused by raised blood pressure, but nevertheless the values in our hypertensive patients in our study group were pathological according to age and sex specific centile charts.^17,18^

The positive correlation between the volume of EAT and cIMT is important because it supports further evaluation of both parameters, which could be obtained during single MRI examination, valuable especially in children with AH. On the other hand, MR imaging is an expensive modality, which is not regularly available in everyday clinical practice. It could be interesting to correlate the results derived from US examination of EAT and cIMT thickness with MR derived values. In case of good correlation, the thickness of cIMT and the volume of EAT could be evaluated in routine examination of children with diagnosed AH, or even in presumably healthy children with certain risk factors (for example family history of AH) in order to assess their cardiovascular risk. This is one further research opportunity which emerged in our study.

One limitation of our study is the small sample size, which affects the generalizability of our findings. The calculated AUC for both predictors of hypertension (cIMT and EATV) was significantly high and might have been slightly influenced by the small sample size, therefore the general applicability of our results is questionable. Further studies with larger cohorts including children with different BMI and diverse ethnic groups should be considered for external validation in order to confirm these predictive values. Another limitation is that we did not collect comprehensive blood and urine samples to obtain laboratory values across the study group. Including these measures, such as lipid levels, blood sugar, plasma renin, serum electrolytes, catecholamine, metanephrine in 24-hour urine and plasma, adrenocorticotropic hormone, thyroid-stimulating hormone, free triiodothyronine, free thyroxine, and plasma cortisol levels, could provide further correlations with EAT and cIMT.

Further studies correlating healthy and hypertensive children with different body types, ethnical background and lifestyle habits could offer further insights into EAT composition, volume, and distribution and correlating findings with cIMT could contribute to effective cardiovascular risk stratification in children.

## Conclusions

Increased EATV and cIMT were found with MRI in hypertensive children compared to their healthy counterparts.

In children, EATV demonstrated a stronger association with hypertension than cIMT.

EATV emerged as an independent predictor of cIMT.

The results of our study suggest that in hypertensive children, an increased EATV is associated with elevated cIMT, indicating a potential early onset of atherosclerosis. Notably, EATV emerged as an independent predictor of cIMT, with both variables positively correlating with hypertension. It is essential to emphasize that while there is a positive correlation between cIMT and EATV in hypertensive children, this does not explicitly indicate a causal relationship. Rather it suggests an area for further research. Additional studies could determine if larger volumes of EAT in hypertensive youth indeed signify an augmented risk of developing cardiovascular complications in adulthood, potentially through the facilitation of early atherosclerosis.
